# The role of contractile dyssynchrony in pacing-induced cardiomyopathy: detailed assessment using index of contractile asymmetry

**DOI:** 10.1186/s12947-023-00308-6

**Published:** 2023-05-01

**Authors:** Patricia Zerlang Fruelund, Anders Sommer, Søren Lundbye-Christensen, Claus Graff, Peter Søgaard, Sam Riahi, Tomas Zaremba

**Affiliations:** 1 Department of Cardiology, Aalborg University Hospital, Hobrovej 18-22, Aalborg, 9000 Denmark; 2 Department of Clinical Medicine, Aalborg University, Forskningens Hus, Sdr. Skovvej 15, Aalborg, 9000 Denmark; 3 Department of Internal Medicine, Regional Hospital of Randers, Randers, Denmark; 4 Unit of Clinical Biostatistics, Aalborg University Hospital, Sdr. Skovvej 15, Aalborg, 9000 Denmark; 5 Department of Health Science and Technology, Aalborg University, Frederik Bajers Vej 7, Aalborg Øst, 9220 Denmark

**Keywords:** Cardiac pacing, Pacing-induced cardiomyopathy, Dyssynchrony, Contractile asymmetry, Computed tomography, Speckle tracking, Strain rate, Echocardiography

## Abstract

**Aims:**

The pathophysiological effects of chronic right ventricular pacing and the role of right ventricular lead position are not well understood. Therefore, we investigated the association between left ventricular contractile dyssynchrony and pacing-induced cardiomyopathy (PICM) in patients with chronic right ventricular pacing. Furthermore, we assessed the association between right ventricular lead location and left ventricular contractile dyssynchrony.

**Methods:**

This was a retrospective study using data from 153 pacemaker patients with normal (≥ 50%) pre-implant left ventricular ejection fraction (LVEF). Baseline and follow-up echocardiograms were analyzed, and PICM was defined as LVEF < 50% with ≥ 10% decrease in LVEF after pacemaker implantation. Relative index of contractile asymmetry (rICA), a novel strain rate-based method, was calculated to quantify left ventricular contractile dyssynchrony between opposing walls in the three apical views. Right ventricular lead position was categorized into anterior septum, posterior septum, free wall, and apex based on contrast-enhanced cardiac computed tomography.

**Results:**

Forty-seven (31%) developed PICM. Overall contractile dyssynchrony, measured by mean rICA, was higher in the PICM group compared with the non-PICM group (1.19 ± 0.21 vs. 1.03 ± 0.19, p < 0.001). Left ventricular anterior-inferior dyssynchrony, assessed in the apical two-chamber view, was independently associated with PICM (*p* < 0.001). Thirty-seven (24%) leads were implanted anterior septal, 11 (7.2%) posterior septal, 74 (48.4%) apical, and 31 (20.3%) free wall. Left ventricular anterior-inferior dyssynchrony was significantly different between the four pacing lead locations (*p* < 0.01) with the highest rICA observed in the posterior septal group (1.30 ± 0.37).

**Conclusions:**

PICM is significantly associated increased contractile dyssynchrony assessed by rICA. This study suggests that especially left ventricular dyssynchrony in the anterior-inferior direction is associated with PICM, and pacing the right ventricular posterior septum resulted in the highest degree of anterior-inferior dyssynchrony. Quantification of left ventricular dyssynchrony by rICA provides important insights to the potential pathophysiology of PICM and the impact of right ventricular lead position.

**Graphical Abstract:**

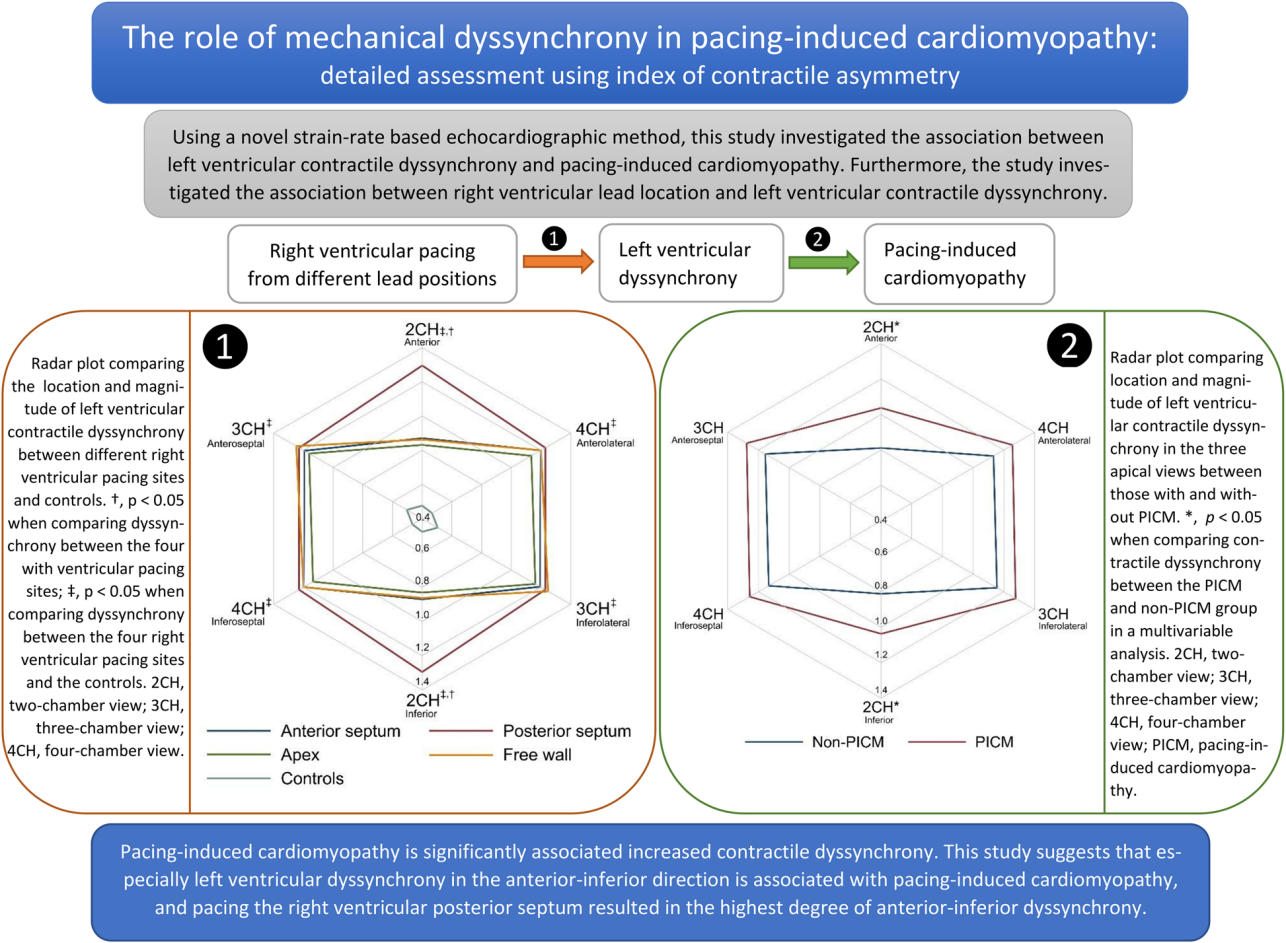

## Background

Driven by the dyssynchronous activation of the myocardium, right ventricular (RV) pacing may have detrimental effects on left ventricular (LV) function resulting in pacing-induced cardiomyopathy (PICM) [1, 2]. It has long been hypothesized that RV septal pacing is superior to non-septal pacing due to the proximity to the specialized conduction system [3]. However, despite extensive research in the past decades, no clinical benefit of RV septal pacing over non-septal pacing has been convincingly demonstrated, and the optimal RV implantation site is still up for debate [4–6].

The RV septum is a complex electroanatomical structure. Pacing from various RV septal regions may result in different activation patterns possibly with no advantage over non-septal pacing [7, 8]. Furthermore, the ideal RV lead position may vary from patient to patient due to individual electroanatomical variations and preexisting myocardial disease affecting the resulting myocardial activation [9]. Therefore, more knowledge is needed to understand the pathophysiological effects of chronic RV pacing and the role of RV lead implantation site.

Speckle tracking echocardiography (STE) has over the years provided important information on myocardial function and mechanical dyssynchrony [10, 11]. However, conventional STE-based methods rely only on a restricted number of strain curves. The recent innovative approach, index of contractile asymmetry (ICA), has been developed to overcome this restriction [12]. Based on STE-derived strain rates, ICA quantifies and localizes LV contractile dyssynchronous activation of entire opposing walls in the three standard apical views [12].

In patients with chronic RV pacing, we investigated the association between LV contractile dyssynchrony and PICM. Furthermore, we assessed the association between RV lead location and LV contractile dyssynchrony.

## Methods

This was a retrospective cohort study including 153 patients who had been implanted with a dual chamber pacemaker at Aalborg University Hospital between March 2012 and May 2020 due to advanced atrioventricular (AV) block. Advanced AV block was defined as second-degree AV block Mobitz type II, 2:1 AV block, higher degree AV block with ≥ 2 consecutive P-waves not conducted and third-degree AV block. All patients had preserved LV ejection fraction (LVEF) (≥ 50%) prior to pacemaker implantation and a high pacing burden (RV pacing ≥ 40%). Patients were excluded if they were unable to attend a study follow-up visit (deceased, terminally ill or moved) or unable or unwilling to provide informed written consent. Furthermore, patients with competing cause of decreased LVEF (severe ischemic heart disease or severe valvular heart disease), device complications with replacement of the RV lead ≥ 3 months after implantation, or contraindications to iodinated contrast agents for cardiac computed tomography (CT) were excluded.

Pre-implant characteristics were obtained by review of electronic medical records. RV pacing percentages were obtained from pacemaker interrogation reports. Participants attended a study-specific follow-up visit including transthoracic echocardiography for assessment of contractile myocardial activation and contrast-enhanced cardiac CT to confirm the RV lead position.

The study was approved by the North Denmark Region (31-1521-103) and study participants signed an informed written consent form. The study was not considered as an interventional study after evaluation by the North Denmark Region Committee on Health Research Ethics and was therefore exempt from requiring formal ethical approval.

### Echocardiography and index of contractile asymmetry

Pre-implant and follow-up echocardiograms were obtained and analyzed. ECG-gated transthoracic two-dimensional echocardiography was performed during RV pacing at study follow-up. Standard apical two-chamber (2CH), three-chamber (3CH), and four-chamber (4CH) images were acquired using a 2.5 MHz transducer. For each image, three consecutive paced cardiac cycles excluding ectopic beats were stored in a cine-loop format for offline analyses. Images for strain analyses were acquired at a high mean frame rate of 106 ± 18 s^-1^. Analyses were subsequently performed offline using EchoPAC® software (GE Healthcare, Milwaukee, WI). Using transaortic continuous wave Doppler trace, the duration of systole was calculated as the time from QRS onset to aortic valve closure. LVEF was calculated using Simpson’s biplane method by two experienced physicians. PICM was defined as LVEF < 50% with ≥ 10% decrease in LVEF after pacemaker implantation.

**Fig. 1:**
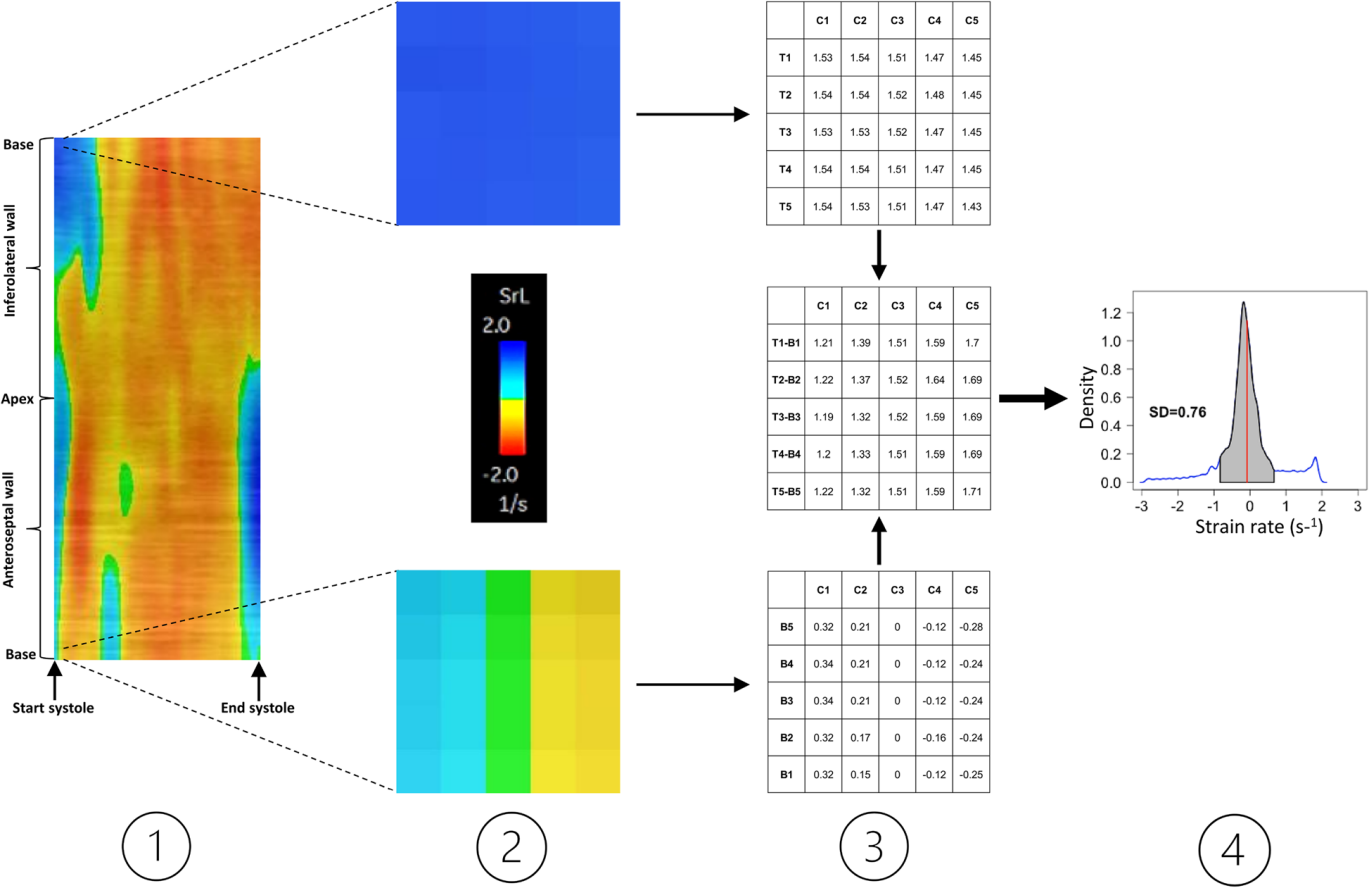
Schematic diagram of calculating ICA. Panel 1: CAMM plot showing the strain rate propagation throughout systole obtained by three-chamber STE-analysis. Panel 2: Zoom on the CAMM plot illustrating the pixels and the color scale used to decode the strain rate values. Panel 3: Extraction of strain rate values from the CAMM plot followed by pairwise subtraction of strain rate values of the opposing corresponding pixels [top rows (T) minus bottom rows (B)] for each column (C), creating a new table containing the strain rate differences (middle table). Panel 4: Density plot of the strain rate differences between the two opposing walls and the corresponding standard deviation (SD) of those differences. This value is referred to as ICA. In this example, ICA in the three-chamber view is 0.76 s^− 1^. CAMM, curved anatomical M-mode; ICA, Index of Contractile Asymmetry; SrL, strain rate; STE, speckle tracking echocardiography

The ICA method is described in detail in the method paper by Zaremba et al. [12]. In short, ICA is calculated from strain rates obtained from the curved anatomical M-mode (CAMM) plots produced by STE-analysis of the standard apical views (Fig. 1). The CAMM plot is a pixilated image showing the strain rate propagation throughout the cardiac cycle. Each pixel represents a strain rate value which is decoded using the color scale provided in the EchoPAC® analysis window. The upper and lower halves of the CAMM plot represents the strain rates between the two opposing walls from apex (center of the CAMM plot) to base (lower and upper extremes of the CAMM plot). Using matrix algebra, the upper and lower part of the table are subtracted from each other by pairwise subtracting the strain rates of the opposing corresponding pixels. This creates a new table containing the strain rate differences for each pair of opposing pixels. Symmetric LV contraction would result in uniform strain rate values between opposing pixels during systole and subtraction would therefore result in strain rate differences close to zero. However, asymmetric LV contraction would result in increased differences in strain rate values between the opposing walls. To quantify the counteractions between the two opposing walls, the standard deviation (SD) of the strain rate differences during systole is calculated for each of the three apical views. This value is referred to as ICA.

While ICA is a measure of contractile dyssynchrony, negative systolic strain rate is related to myocardial contractility [13]. To adjust ICA for the contractile properties of the LV myocardium, ICA was indexed to the mean negative systolic strain rate in the particular apical view. This adjusted ICA was termed relative ICA (rICA). rICA was calculated for each of the three apical views. Hence, rICA 2CH quantifies dyssynchrony between the LV anterior and inferior wall, rICA 3CH between the LV inferolateral and anteroseptal wall, and rICA 4CH between the LV anterolateral and inferoseptal wall. To assess the overall degree of LV dyssynchrony, mean rICA was calculated as the average of rICA in the three apical views.

Furthermore, rICA was calculated from echocardiograms performed in 10 healthy individuals with no history of cardiovascular disease and used as controls.

### Cardiac computed tomography and right ventricular lead position

RV lead position was determined from cardiac CT. A contrast-enhanced CT scan was obtained for all patients. Some patients had a high-quality contrast-enhanced CT scan with clear visualisation of the RV lead tip available at study follow-up (*n* = 42). If no scan was available, a study-specific CT scan was performed at the follow-up visit (*n* = 111). The scans were performed using a second-generation dual source scanner (Siemens Somatom Definition Flash, Siemens Healthcare, Erlangen, Germany). The scans were ECG-synchronised and performed during breath hold at the end of inspiration. Administration of contrast was timed for optimal visualisation of both the right and left ventricle.

The CT scans were analyzed using commercially available DICOM software. The RV lead position was analyzed using a regional approach [14]. The RV lead tip position was determined dividing the RV long-axis cavity into equal thirds (basal, mid, and apical). The RV lead position was then evaluated in the short-axis (three basal segments [anterior septum, posterior septum, and free wall], three mid RV segments [anterior septum, posterior septum, and RV free wall], and two apical segments [septum and free wall]). Subsequently, the RV lead positions were grouped into anterior septum (basal and mid anterior septum), posterior septum (basal and mid posterior septum), apex (apical septum) and free wall (basal, mid, and apical).

### Statistics

Continuous values are reported as mean and SD or median and interquartile range as appropriate. Categorical values are expressed as absolute numbers and percentages. One-way analysis of variance was used for analyzing the differences in rICA between groups. To accommodate potential non-normality and variance inhomogeneity, standard errors were calculated using bootstrap with 5000 replications. The association between two continuous variables was evaluated using regression and Pearson’s rho. Receiver operating characteristics (ROC) analysis was applied to assess the predictive performance of rICA. Cutoff values were calculated corresponding to the Youden index. Modified Poisson regression with robust variance estimation was used to estimate relative risks (RR) between groups. Two-sided tests were applied and *p* < 0.05 was considered statistically significant. All statistical analyses were performed using STATA version 17.

## Results

During follow-up, 47 (31%) developed PICM and an overall reduction in LVEF of 8% ± 10% was observed. Pre-implant characteristics at time of pacemaker implantation are shown in Table 1. There was a median follow-up of 3.1 years (1.9–4.8) with no difference between the PICM and non-PICM group (3.1 [1.8–4.5] versus 3.1 [2.1–4.9], *p* = 0.3). There was a median pacing percentage of 96.5% (85.8–99.8) with no difference between the PICM and non-PICM group (95.4% [85.7–100.0] versus 97.0% [85.9–99.5], *p* = 0.9).

**Table 1:** Pre-implant characteristics

	All subjects (*n* = 153)	PICM (*n* = 47)	Non-PICM (*n* = 106)	*P* value
Age (years)	72 (65–77)^a^	72 (67–76)^a^	72 (64–77)^a^	0.91
Male	103 (67)	35 (74)	68 (64)	0.26
LVEF (%)	60 ± 4	59 ± 4	61 ± 3	0.01
QRS duration	119 ± 30	125 ± 32	117 ± 29	0.14
Comorbidities				
Ischemic heart disease	9 (6)	2 (4)	7 (7)	0.72
Valvular heart disease	12 (8)	4 (9)	8 (8)	1.0
Atrial fibrillation	15 (10)	2 (4)	13 (12)	0.15
Hypertension	106 (69)	35 (74)	71 (67)	0.45
Diabetes	35 (23)	16 (34)	19 (18)	0.04
History of smoking	65 (43)	24 (51)	41 (39)	0.16
eGFR (ml/min/1.73 m^2^)	75 ± 15	72 ± 15	76 ± 16	0.17

Continuous values are reported as mean ± SD or median^a^ (interquartile range) as appropriate. Categorical values are expressed as numbers (%). *LVEF* Left ventricular ejection fraction, *eGFR* Estimated glomerular filtration rate

### Relative index of contractile asymmetry and pacing-induced cardiomyopathy

**Fig. 2:**
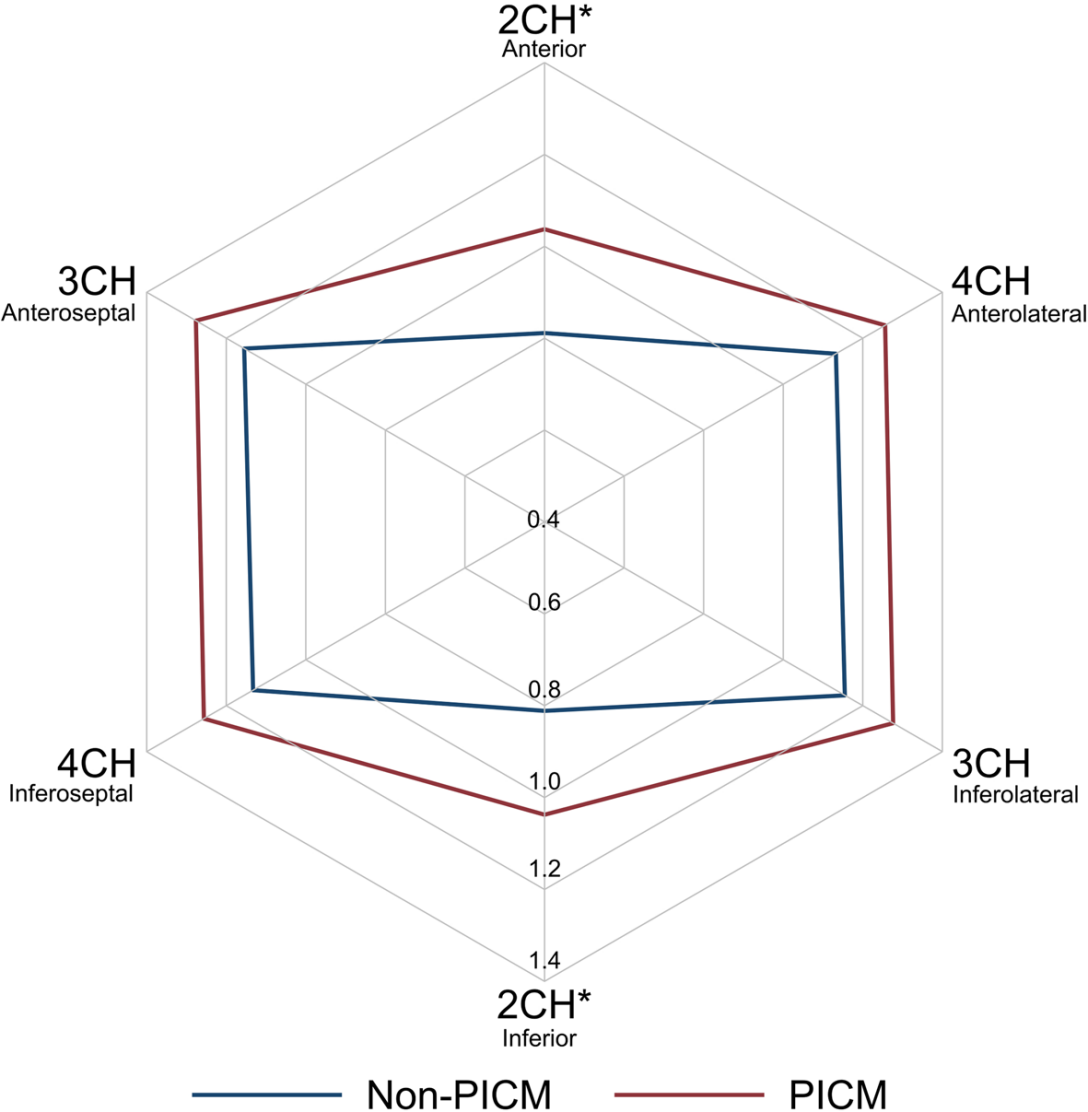
Illustration of the degree and location of LV dyssynchrony. The radar plot shows the 2CH, 3CH and 4CH rICA values for the PICM group (red) and non-PICM group (blue). rICA, relative index of contractile asymmetry; PICM, pacing-induced cardiomyopathy; 2CH, two-chamber; 3CH, three-chamber; 4CH, four-chamber; *, *p* < 0.05 when comparing rICA between the PICM and non-PICM group in a multivariable analysis

The radar plot in Fig. 2 illustrates the degree and location LV dyssynchrony assessed by rICA for the PICM and non-PICM group. For both groups, rICA was highest in the 3CH view and lowest in 2CH view. While the pattern of rICA was similar in the two groups, the PICM group exhibited consistently higher rICA in the three views (Table 2). The greatest difference in rICA was seen in the 2CH view. In multivariable analysis including rICA in all three views, only rICA 2CH was independently associated with PICM (*p* < 0.001). Therefore, rICA 2CH was chosen for further analyses.

**Table 2:** rICA for the PICM and non-PICM group

	PICM (*n* = 47)	Non-PICM (*n* = 106)	rICA difference (95% CI)	*P* value
rICA 2CH	1.04 ± 0.30	0.81 ± 0.25	0.23 (0.14–0.32)	< 0.001*
rICA 3CH	1.28 ± 0.24	1.15 ± 0.27	0.12 (0.03–0.21)	0.005*
rICA 4CH	1.26 ± 0.23	1.13 ± 0.24	0.12 (0.04–0.20)	0.002*
rICA mean	1.19 ± 0.21	1.03 ± 0.19	0.15 (0.10–0.22)	< 0.001*

*rICA* Relative Index of Contractile Asymmetry, *2CH* Two-chamber, *3CH* Three-chamber, *4CH* Four-chamber

*P < 0.05

ROC analysis of rICA 2CH for prediction of having PICM yielded an area under the curve (AUC) of 0.73 [95% confidence interval (CI) 0.65–0.82]. A cutpoint of rICA 2CH of 0.90 had a 70% sensitivity, a 72% specificity, a positive predictive value (PPV) of 52%, and a negative predictive value (NPV) of 84% for PICM. rICA 2CH ≥ 0.90 yielded a RR of 3.4 (95% CI 2.0-5.8) for having PICM. Changing the outcome to LVEF ≤ 40% yielded an AUC of 0.82 (95% CI 0.74–0.90). Looking at follow-up LVEF ≤ 40%, a 0.90 rICA 2CH cutpoint had a 90% sensitivity, a 66% specificity, a PPV of 29%, and a NPV of 98%. rICA 2CH ≥ 0.90 yielded an RR of 12.9 (95% CI 3.1–53.7) for having a follow-up LVEF ≤ 40%.

### Correlation between contractile dyssynchrony and change in left ventricular ejection fraction

**Fig. 3:**
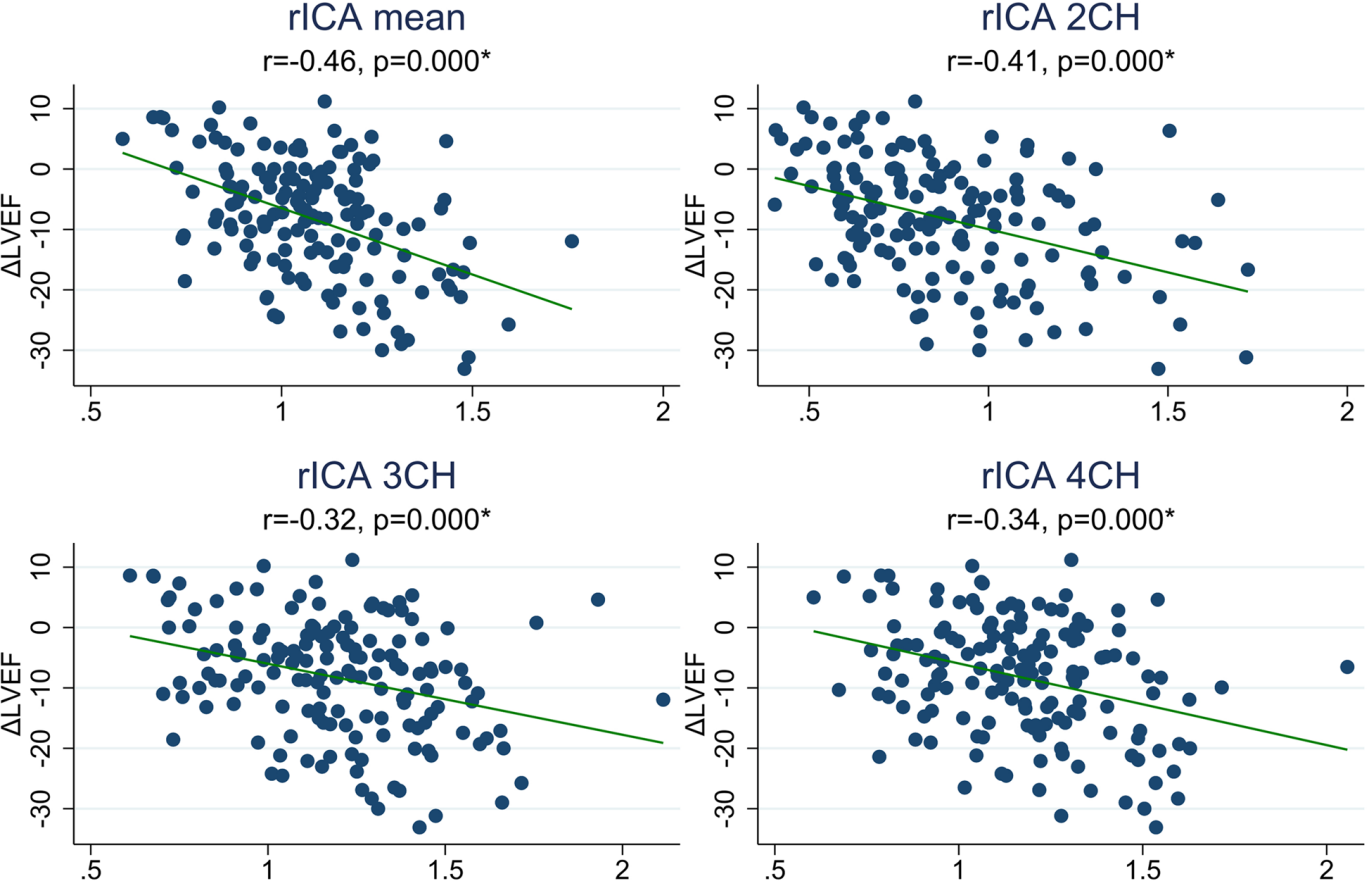
Association between change in LVEF and rICA. rICA, relative index of contractile asymmetry; ΔLVEF, change in left ventricular ejection fraction from baseline to end of follow-up; r, Pearson’s r; *, *p* < 0.05

Figure 3 shows the association between rICA and change in LVEF from baseline to follow-up. The strongest association between change in LVEF and rICA was seen for rICA mean and rICA 2CH. A 1.0 increase in rICA mean was associated with an absolute LVEF reduction of 22% (95% CI 15–29%). A 1.0 increase in rICA 2CH was associated with an absolute LVEF reduction of 14% (95% CI 9–19%). The association between rICA and change in LVEF was significant in all three views (*p* = 0.000 for each view). The linear regression slopes were not significantly different between the three apical views (*p* = 0.80). However, the regression slope for mean rICA was significantly steeper and the correlation higher compared jointly with the regression slopes for the three apical views (*p* = 0.003).

### Right ventricular lead position and contractile dyssynchrony

Determined by cardiac CT, the RV lead was implanted throughout the RV endocardium with 37 (24%) located on the anterior septum, 11 (7.2%) located on the posterior septum, 74 (48.4%) located in the apical region and 31 (20.3%) located on the free wall.

Mean rICA was borderline significantly different between the four RV lead groups (Table 3). In the three apical views separately, rICA was significantly different between the groups in the 2CH view but not in the 3CH view or 4CH view. The highest mean rICA and highest rICA 2CH was observed in the posterior septum group. Compared with the RV paced patients, the controls demonstrated consistently lower rICA.

**Table 3:** rICA for each RV lead group and controls

	Anterior septum (*n* = 37)	Posterior septum (*n* = 11)	Apex (*n* = 74)	Free wall (*n* = 31)	*P* value^†^	Controls	*P* value^‡^
rICA 2CH	0.87 ± 0.23	1.30 ± 0.37	0.83 ± 0.26	0.86 ± 0.24	0.001*	0.48 ± 0.17	< 0.001*
rICA 3CH	1.19 ± 0.26	1.23 ± 0.31	1.16 ± 0.24	1.25 ± 0.31	0.55	0.50 ± 0.15	< 0.001*
rICA 4CH	1.20 ± 0.27	1.23 ± 0.28	1.14 ± 0.20	1.20 ± 0.27	0.36	0.47 ± 0.13	< 0.001*
rICA mean	1.09 ± 0.20	1.25 ± 0.26	1.04 ± 0.18	1.10 ± 0.22	0.04*	0.48 ± 0.14	< 0.001*

*rICA* Relative Index of Contractile Asymmetry, *2CH* Two-chamber, *3CH* Three-chamber, *4CH* Four-chamber

†Comparing rICA between the four RV lead groups

‡Comparing rICA between the four RV lead groups and the controls

*P < 0.05

**Fig. 4:**
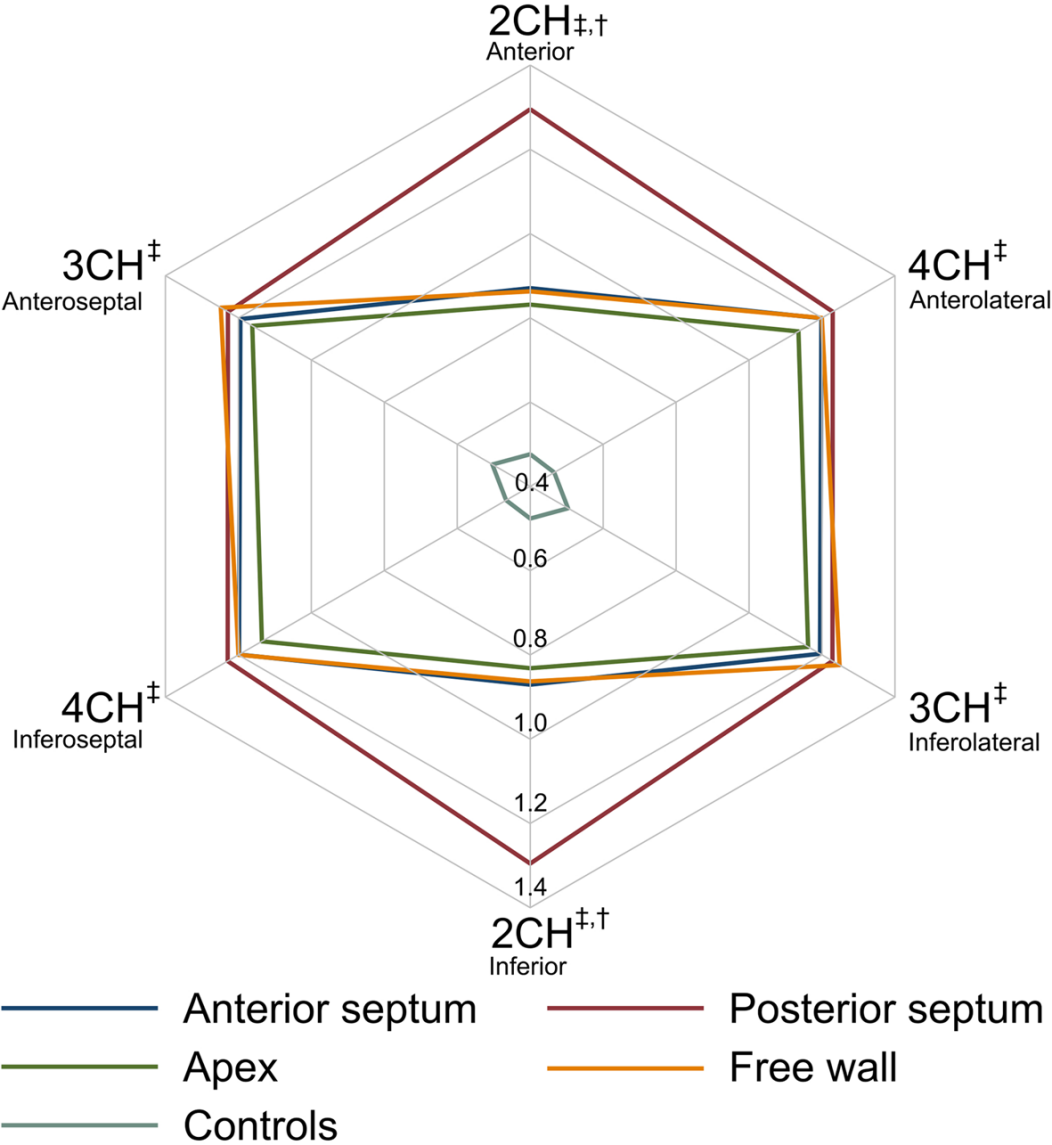
Radar plot illustrating the degree and location of LV dyssynchrony by showing the 2CH, 3CH and 4CH rICA for each of the four RV lead groups and the controls. rICA, relative Index of Contractile Asymmetry; 2CH, two-chamber; 3CH, three-chamber; 4CH, four-chamber; †, p < 0.05 when comparing rICA between the four RV lead groups; ‡, p < 0.05 when comparing rICA between the four RV lead groups and controls

The radar plot in Fig. 4 shows the rICA in the three apical views for the four RV lead groups and the controls. Similar rICA patterns were observed in the anterior septum group, the apex group, and the free wall group with rICA being lowest in the 2CH view and highest in the 3CH and 4CH views. In contrast, rICA was consistently high in all three views in the posterior septum group. In the control group, rICA was consistently low in all three views.

## Discussion

In this study, using rICA for detailed assessment of LV dyssynchrony in chronically RV paced patients, we found: ① Both magnitude and location of LV dyssynchrony were significantly associated with PICM and reduction in LVEF. ② Anterior-inferior LV dyssynchrony assessed by rICA in the 2CH view was highly predictive of having PICM. ③ RV lead location was significantly associated with both magnitude and location of LV dyssynchrony with the highest degree of LV dyssynchrony observed in those paced from the posterior septum.

### The role of contractile dyssynchrony in pacing-induced cardiomyopathy

This study supports that LV mechanical dyssynchrony is a driving factor in PICM [1, 15]. It seems that location of LV dyssynchrony plays a significant role in PICM. Though rICA in each of the apical views was significantly associated with PICM in univariable analysis, only rICA 2CH was independently associated with PICM. Furthermore, in subsequent ROC analyses, rICA 2CH proved predictive of having PICM. Thus, it seems that especially dyssynchrony in the LV anterior-inferior direction is potentially detrimental. Hypothetically, pacing the RV posterior septum may induce a more detrimental activation sequence with early activation of the LV inferoseptal and inferior wall with pre-stretch of the anterior wall [15]. This initial activation may not generate enough force to open the aortic valve, and the initial myocardial work is wasted. Subsequently, the pre-stretched LV anterior wall is activated, forcing the blood flow away from the anteriorly positioned LV outflow tract and back towards the prematurely relaxed inferior region. Ultimately, this may result in lower myocardial efficiency, premature closure of the aortic valve and decreased cardiac output [7, 8, 15]. In contrast, pacing anteriorly may result in an activation sequence mimicking intrinsic activation, even though the overall activation may be slower. This hypothesis should be investigated in future studies. Additionally, the significance of rICA measured between the anterior and inferior walls may also be explained by more widely distributed LV dyssynchrony also detectable in the 2CH view. Supporting this, the regression slope for mean rICA and change in LVEF was significantly steeper when compared jointly with rICA 2CH, 3CH, and 4CH. However, the linear correlation coefficients between change in LVEF and the different rICA measures were weak to moderate. Thus, decrease in LVEF cannot solely be explained by contractile dyssynchrony at follow-up. Measurement errors regarding both LVEF and rICA may have contributed to this. Furthermore, other factors besides contractile dyssynchrony may have influenced change in LVEF after pacemaker implantation. This was not further investigated in this study.

Different echocardiographic techniques to assess LV mechanical dyssynchrony have been developed over the years. Early on, methods like M-mode and pulsed Doppler have been used to assess wall motion delay or delay in RV an LV outflow as indicators of dyssynchrony [16]. Later, tissue Doppler imaging and STE allowed for development of strain-based methods to quantify dyssynchrony including time-to-peak and cross-correlation analysis [10, 16]. However, traditional STE-based or TDI-based methods rely on a restricted number of curves, providing only a crude estimation of LV dyssynchrony [10, 17, 18]. Thus, their contribution to a deeper understanding of cardiac mechanics during RV pacing is limited. ICA is an elaborate STE-based method using > 160 data lines from each myocardial wall obtained from the three apical views. This has provided the opportunity to assess, in detail, regional differences in contractile dyssynchrony between entire opposing walls. The ICA method has previously been validated by members of this study group using prospective data from heart failure patients undergoing cardiac resynchronization therapy (CRT) device implantation [12]. The study found that both location and degree of pre-CRT dyssynchrony was predictive of CRT response. Furthermore, the ICA method was found to be robust, demonstrating a high intraobserver agreement with an intraclass correlation coefficient of 0.89 (95% CI 0.82–0.93).

Building on the initial experiences with ICA, we indexed ICA to the mean negative systolic strain rate in this study to make the assessment of LV contractile dyssynchrony independent of myocardial contractility. This yielded a more balanced parameter of dyssynchrony in a pacemaker population with heterogenous myocardial contractility. Using rICA, we comprehensively investigated both the degree and location of LV mechanical dyssynchrony, thus providing new insights to the pathophysiology of RV pacing.

### Right ventricular lead position and contractile dyssynchrony

Generally, we found that pacing the anterior septum, apex and free wall resulted in similar activation patterns with rICA being lowest in the 2CH view and highest in the 3CH and 4CH view. In contrast, the posterior septum group showed consistently high rICA in all views. The differences in LV activation patterns between the RV lead positions may be explained by differences in recruitment of the specialized conduction system as well as the RV lead position relative to the LV. In this study, the apical and free wall groups were not further divided into anterior and posterior. However, looking at the distribution of the septal leads, most leads were located anteriorly. Thus, it is likely that the distribution is similar in the apical and free wall groups with the majority of the leads being implanted anteriorly. Therefore, those three categories may all primarily induce early anterior LV activation resulting in similar LV activation patterns. This is merely hypothetical and should be investigated in future studies.

In a previous study, using the same RV lead categories, we found no significant association between RV lead position and risk of PICM [2]. Meanwhile, the present study shows an association between both RV lead position and the degree of LV dyssynchrony and an association between the degree of LV dyssynchrony and PICM. This suggests, that although RV lead position may affect the pattern and degree of dyssynchronous activation other factors affecting LV dyssynchrony and cardiac function must contribute to the response to cardiac pacing and risk of PICM.

The potential detrimental consequence of pacing the RV posterior region is in line with a study by Vančura et al., who investigated the acute effects of RV pacing from 18 different RV locations on invasive LV hemodynamic measures [7]. They found consistently that pacing in all posterior RV segments, free wall or septal, resulted in worse hemodynamic responses. To our knowledge, this is the first study looking at the chronic effects of pacing the RV posterior septum. Thus, exposing a potential risk of worse outcome when aiming for septal implantation.

The vast majority of clinical studies searching for the optimal RV lead position, have applied a simplistic, often binary, categorization of RV lead locations. However, any categorical RV location may demonstrate large electroanatomical variations [7, 19]. Consequently, this may have contributed to the neutral results from studies investigating the effects of RV lead position on clinical outcomes using a binary classification of exposure groups such as septal versus non-septal [5, 20, 21]. These studies may unintentionally have clustered patients with completely different responses to RV pacing. In this study, with further dividing the RV lead position into four categories, we found a significant difference in the dyssynchrony induced by different RV lead locations. However, all RV pacing sites resulted in significantly more dyssynchrony compared with the controls. Thus indicating, that RV pacing, irrespective of pacing site, will inevitably induce some degree of potentially harmful dyssynchronous activation perhaps only mitigated by CRT or conduction system pacing.

### Limitations

This was a single-center retrospective study with a relatively small sample size limiting its power, especially when dividing the RV lead position into four categories for comparison. Also, as study inclusion was done after pacemaker implantation, there is a risk of selection bias. The study did not consider clinical endpoints which would have contributed to a more comprehensive evaluation of the consequences of RV pacing. Assessment of dyssynchrony was done in a follow-up echocardiogram after a median of 3.1 years of follow-up and baseline assessment of RV paced dyssynchrony was not available. Consequently, this study is not predictive of developing PICM but should be considered more descriptive when concluding on the effects of chronic RV pacing on LV mechanical dyssynchrony. However, a substudy of Protect-PACE, a randomized controlled trial comparing RV apical and non-apical pacing, demonstrated consistent levels of LV dyssynchrony with no significant changes during the 2-year study follow-up period [18]. Therefore, it is likely that the follow-up assessment in this study reflects both the acute and chronic changes in LV mechanical dyssynchrony. This is merely hypothetical and rICA should be investigated as a potential tool to identify patients at risk of developing PICM in future prospective studies.

## Conclusions

Results from this first study, applying rICA to assess LV mechanical dyssynchrony in chronically RV paced patients, revealed that PICM is significantly associated increased rICA. This study suggests that especially LV dyssynchrony in the anterior-inferior direction is associated with PICM and pacing the RV posterior septum resulted in the highest degree of anterior-inferior dyssynchrony. Quantification of LV dyssynchrony by rICA provides important insights to the pathophysiology of PICM and the impact of RV lead position.

## Data Availability

Data underlying this article is not publicly available out of consideration for the study participants. Relevant data can be made available upon reasonable request to the corresponding author.

## Abbreviations

AUC: Area under the curve
AV: Atrioventricular
CAMM: Curved anatomical M-mode
CI: Confidence interval
CT: Computed tomography
eGFR: Estimated glomerular filtration rate
ICA: Index of contractile asymmetry
LV: Left ventricle
LVEF: Left ventricular ejection fraction
NPV: Negative predictive value
PICM: Pacing-induced cardiomyopathy
PPV: Positive predictive value
rICA: Relative index of contractile asymmetry
ROC: Receiver operating characteristics
RV: Right ventricle
SD: Standard deviation
STE: Speckle-tracking echocardiography
2CH: Two-chamber
3CH: Three-chamber
4CH: Four-chamber
